# In Vivo Long Head of the Biceps Tendon Stiffness Varies with Forearm Position During Active Contraction: Implications for Personalized Rehabilitation After SLAP Lesions

**DOI:** 10.3390/jpm16040194

**Published:** 2026-04-01

**Authors:** Zade Pederson, Hugo Giambini

**Affiliations:** Department of Biomedical Engineering and Chemical Engineering, The University of Texas at San Antonio, San Antonio, TX 78249, USA

**Keywords:** long head of the biceps tendon, shear wave elastography, tendon loading, position, personalized medicine, precision rehabilitation, patient-specific biomechanics

## Abstract

**Background/Objectives**: Type II superior labrum anterior–posterior (SLAP) lesions of the long head of the biceps (LHB) tendon are associated with excessive tendon loading and are commonly treated surgically using SLAP repair, tenotomy, or tenodesis. These procedures alter musculotendinous length and loading and may affect functional outcomes, including forearm supination strength. Appropriate restoration of tendon tension is critical for favorable muscle adaptation and recovery. Shear wave elastography (SWE) is a non-invasive imaging technique capable of quantifying tissue stiffness as a surrogate for in vivo musculotendinous tension. This study aimed to characterize LHB tendon tension across forearm positions and loading conditions to improve the understanding of functional tendon loading relevant to postoperative activation and rehabilitation. **Methods**: In this controlled laboratory study, thirteen healthy female volunteers without shoulder pathology were assessed using SWE with the elbow positioned at 90° flexion. LHB tendon tension was measured in forearm pronation and supination under passive, active (unresisted), and weighted conditions. Paired t-tests were used to compare forearm positions within each loading condition. **Results**: LHB tendon tension was significantly greater during active and weighted conditions compared with passive loading in the pronated position (*p* < 0.05). During active contraction, tendon tension was significantly lower in supination than pronation (*p* < 0.05), whereas no positional differences were observed under passive or weighted conditions. Relative to passive loading, tendon tension increased by approximately 18.2% and 89.2% in supination, and 67.0% and 97.9% in pronation during active and weighted conditions, respectively. **Conclusions**: Forearm position selectively influences LHB tendon tension during active, unresisted contraction. Forearm orientation affected LHB tendon stiffness primarily during active, unweighted contraction, where pronation resulted in higher stiffness than supination. On the other hand, stiffness outcomes measured during passive and weighted positions were comparable between forearm orientations, indicating that positional effects are most evident when tendon loading is primarily muscle-driven. These findings highlight the relevance of forearm positioning during early postoperative activation and provide normative in vivo reference data to inform personalized rehabilitation strategies and future investigations of postoperative tendon loading following SLAP lesion treatment.

## 1. Introduction

The long head of the biceps (LHB) tendon is integral in shoulder stabilization and forearm supination, serving as a dynamic stabilizer to glenohumeral joint function [1,2]. The LHB originates from the supraglenoid tubercle of the scapula and passes intraarticularly before traveling through the intertubercular sulcus or bicipital groove of the humerus, a region that acts as a mechanical guide for tendon stability during dynamic upper limb motion [3]. Biomechanical studies show that the LHB tendon undergoes complex multiaxial deformations during shoulder motion and plays a crucial role in stress absorption during overhead functional activities [4,5,6]. Injuries to the LHB tendon, particularly Type II Superior Labrum Anterior–Posterior (SLAP) lesions, can result in pain, instability, and overall impaired performance [1,7,8,9]. Type II SLAP lesions are characterized by detachment of the superior glenoid labrum at the insertion of the LHB tendon, with higher prevalence in overhead athletes [1,9].

SLAP lesions represent a clinically relevant source of shoulder dysfunction and are reported in approximately 6–26% of patients undergoing shoulder arthroscopy, depending on the population studied and associated pathology [10]. Management of Type II SLAP lesions typically involves surgical intervention using one of three methods: SLAP repair, tenotomy, or tenodesis [11,12]. These procedures have shown variable clinical outcomes, with persistent postoperative pain, muscle atrophy, or decreased supination strength reported in some patients [7,13,14,15]. SLAP repair aims to anatomically restore the labral–biceps anchor complex through suture-based techniques [16], while tenotomy and tenodesis involve the release of the LHB tendon, with tenodesis including reattachment to the humerus using various fixation strategies [17]. Tenodesis is often preferred in younger or active individuals due to its reduced incidence of the Popeye deformity, a cosmetic defect more common after tenotomy [17,18,19,20]. Although tenodesis offers cosmetic advantages, appropriate restoration of physiological tendon tension is critical for functional recovery and for minimizing postoperative complications [13,21]. A recent retrospective analysis reported that approximately 11,000 SLAP repairs, 10,000 arthroscopic tenodesis, and 9500 open tenodesis were performed between 2003 and 2023, reflecting substantial volume associated with these injuries [22]. Importantly, patients undergoing SLAP repair have been reported to have increased risk of revision surgery, while patients undergoing biceps tenodesis experienced higher rates of postoperative pain and shoulder stiffness [22].

Tendon tension directly impacts the muscle-tendon unit’s length relationship, a key determinant of optimal force production. Improper reattachment length can lead to sarcomere adaptation to reestablish equilibrium, which may result in suboptimal actin–myosin overlap and decreased contractile force [23,24,25,26,27,28]. Both overtightening and slack reattachment may lead to altered shoulder and forearm biomechanics, and persistent symptoms [13,19]. Despite its clinical relevance, normative in vivo data on LHB tendon tension under varying physiological conditions remain limited, restricting our understanding of the functional loading environment experienced by the tendon following repair [16,29].

Shear wave elastography (SWE) is an ultrasound-based technique that noninvasively quantifies soft tissue stiffness and provides a reliable surrogate for in vivo tendon tension. While SWE has been used in basic science and clinical research to assess tendinopathies of the Achilles [30] and supraspinatus tendons [31], and assess muscle properties [32,33,34,35], among some, its application to the LHB tendon remains underexplored. Furthermore, the effect of arm position (i.e., forearm pronation vs. supination) on LHB tension has not been systematically studied, leaving a gap in our ability to interpret postoperative function and early functional loading following SLAP repair or tenodesis. This is of particular interest to develop tailored treatment and rehabilitation strategies to individual biomechanical and functional profiles. However, such approaches require quantitative in vivo standards that describe how tissues behave under different physiological conditions. The lack of normative, condition-specific tendon loading data limits the ability to individualize rehabilitation following shoulder injury or surgical repair. Thus, establishing baseline stiffness profiles of the LHB tendon across functional conditions may therefore provide a critical foundation for personalized, data-driven clinical decision-making.

This study aimed to characterize in vivo stiffness profiles of the LHB tendon in asymptomatic individuals using SWE across multiple loading conditions and forearm positions. Establishing these normative baseline descriptors provides a foundation for future comparisons with individuals who have undergone surgical repair for Type II SLAP lesions. Ultimately, these data may inform postoperative strategies and support future work investigating how surgical fixation parameters and rehabilitation protocols influence functional tendon loading and recovery.

## 2. Materials and Methods

This was a controlled laboratory study using a repeated-measures experimental design to evaluate in vivo stiffness of the LHB tendon under different forearm positions and loading conditions using SWE. Each participant underwent measurements across all experimental conditions, allowing within-subject comparisons of tendon stiffness across forearm orientations and loading states. Data were collected from February to June 2025.

Thirteen female volunteers (mean age: 21 ± 2.5 years; weight 57.6 ± 7.9 kg; height 160.9 ± 4.3 cm) without prior surgery or functional limitations of the dominant arm participated in this study. Participants were recruited from a university population and participated voluntarily. Inclusion criteria included age between 18 and 65 years, and no history of shoulder pain or shoulder pathology. Exclusion criteria included BMI > 32, prior shoulder surgery, multiple sclerosis, myopathies, prior shoulder trauma, neuromuscular conditions (e.g., myasthenia gravis) or other muscle and tendon disorders. The study protocol was approved by our Internal Review Board (IRB # 19-053; 12 December 2018). Written informed consent was obtained from all participants prior to data collection and no personally identifiable information was recorded. All procedures were conducted in accordance with the ethical principles outlined in the Declaration of Helsinki.

**Figure 1:**
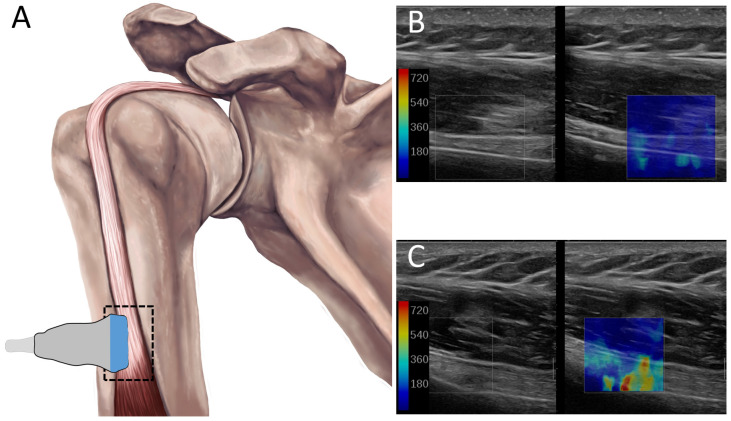
(**A**) Schematic of ultrasound probe placement over the long head of the biceps (LHB) tendon. (**B**,**C**) Representative ultrasound shear wave elastography (SWE) images showing the tendon and corresponding stiffness maps for the (**B**) passive and (**C**) weighted conditions. Colors represent varying degrees of stiffness in the tissue. The color bar indicates a corresponding stiffness range.

**Figure 2:**
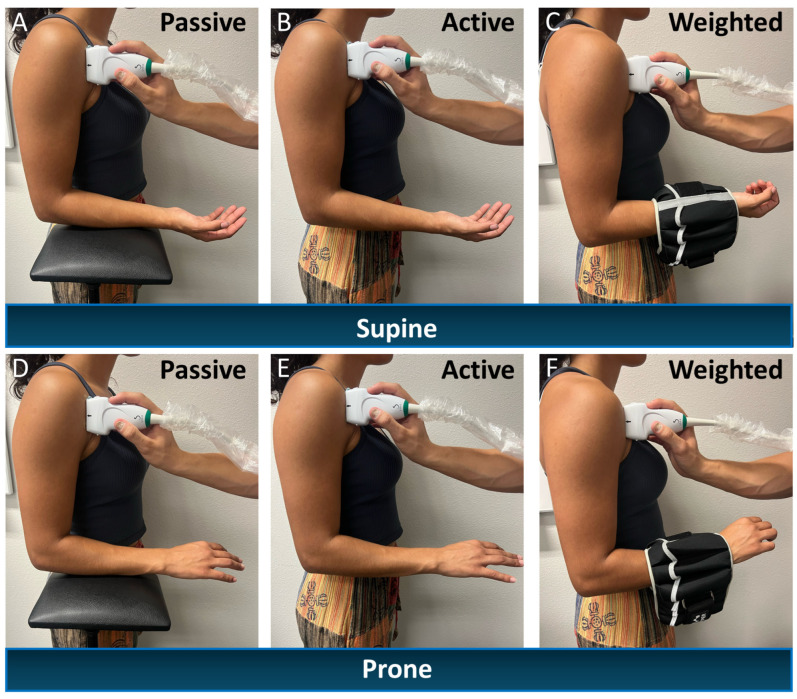
Representative subject illustrating ultrasound shear wave elastography (SWE) measurements obtained in supinated (**A**–**C**) and pronated (**D**–**F**) forearm positions under three conditions: passive (relaxed muscle; **A**,**D**), active (voluntary, unresisted contraction; **B**,**E**, unresisted), and weighted (external load applied; **C**,**F**).

SWE-measured stiffness (kPa, kilopascals) of the LHB tendon, as a surrogate for tension, were obtained using a Mach-30 ultrasound system equipped with a 10–2 MHz linear array probe (Supersonic Imagine, Aix-en-Provence, France). SWE images of the LHB tendon were acquired distal to the bicipital groove in both pronated and supinated forearm positions, under three conditions: passive, active, and weighted. For all conditions, participants maintained their dominant arm at a 90° elbow flexion. During passive assessments, the forearm was supported on a stand to eliminate voluntary muscle activation. The stand was removed for the active and weighted conditions, and participants were instructed to maintain the same position. In the weighted condition, a weight equal to 1% of the participant’s total body mass was suspended from the wrist. For each condition, three SWE images were collected. Regions of interest (ROIs) were consistently placed within the tendon, proximal to the point of intramuscular insertion, to ensure uniformity in measurement location. The average stiffness (kPa) value across the three ROIs for each condition was used for analysis. Ultrasound probe placement and representative SWE images are shown in Figure 1. Experimental setup for the passive, active, and weighted conditions are illustrated in Figure 2.

### Statistical Analysis

Shear wave elastography (SWE)-measured stiffness values (kPa) were compared between the supinated and pronated forearm positions within each experimental condition (passive, active, and weighted). Because the same participants were tested in both positions, paired t-tests were performed to evaluate differences in stiffness across forearm positions for each condition separately. Normality of the data distribution was verified using the Shapiro–Wilk test prior to applying parametric testing. Percent change was calculated for each participant individually [(post − pre)/pre × 100], and the resulting percent changes were then average to obtain the group mean ± standard error (SE). This approach preserves inter-individual variability and avoids bias associated with calculating percent change from group-averaged values. Results are reported as mean ± SE, with statistical significance set at *p* < 0.05.

## 3. Results

Average LHB tendon stiffness outcomes demonstrated position- and condition-dependent variations. In the supinated forearm position, mean ± SE stiffness were 155 ± 20 kPa in the passive condition, 177 ± 27 kPa in the active condition, and 280 ± 42 kPa in the weighted condition. In the pronated forearm position, mean stiffness values were 155 ± 16 kPa (passive), 247 ± 39 kPa (active), and 292 ± 38 kPa (weighted).

**Figure 3:**
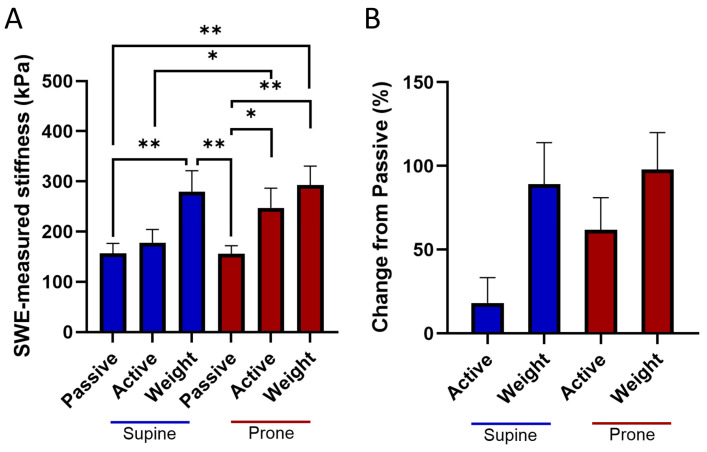
(**A**) Comparisons of ultrasound shear wave elastography (SWE)-measured stiffness across conditions and forearm positions, showing significant increases within supinated and pronated positions, as well as differences between positions (*: *p* < 0.05; **: *p* < 0.01). (**B**) Percentage change in long head of the biceps (LHB) tendon stiffness across conditions, illustrating the effects of muscle activation and external loading in both supinated and pronated positions.

In the supinated position, stiffness significantly increased from passive to weighted (*p* < 0.01). Within the pronated position, stiffness significantly increased from passive to active (*p* < 0.05) and passive to weighted (*p* < 0.01). Comparisons across forearm positions showed that supinated passive was significantly lower than pronated weighted (*p* < 0.01), supinated active was significantly lower than pronated active (*p* < 0.05), and supinated weighted was significantly greater than pronated passive (*p* < 0.01) (Figure 3A).

Percentage changes in stiffness further illustrate the effects of muscle activation and external loading on LHB tendon tension. In the supinated position, transitioning from passive to active contraction increased stiffness by 18.2 ± 14.6%, while the addition of external load (weighted condition) led to increases of 89.2 ± 23.8% relative to passive and a 67.0 ± 11.9% relative to active. In the pronated position, activation increased stiffness by 61.8 ± 18.5% relative to passive, and loading led to increases of 97.9 ± 21.1% relative to passive and 32.0 ± 12.8% relative to active (Figure 3B).

## 4. Discussion

The present study is the first to quantify LHB tendon stiffness across a range of forearm orientations and loading conditions using SWE in a healthy female population. The results provide novel in vivo baseline data on how musculotendinous tension within the LHB varies with pronation and supination at different activation states. Importantly, the results demonstrate that while passive and externally resisted conditions show minimal differences between forearm positions, pronation selectively increases tendon stiffness during active unresisted contraction.

The present findings have important implications for the functional use of the biceps following treatment of Type II SLAP lesions, particularly during early postoperative activation and low-load rehabilitation rather than during resting or high-load conditions. Prior in vivo and clinical studies have demonstrated the importance of restoring appropriate tension to the LHB tendon during repair, as improper tension has been associated with reduced strength and altered joint biomechanics [13]. However, few studies have provided quantitative in vivo tension values under physiologic conditions. This study fills that gap by establishing reproducible SWE-derived stiffness values across a range of static loading conditions, suggesting that SWE may serve as a valuable tool for characterizing functional musculotendinous loading and monitoring postoperative adaptation.

Findings from this study suggest that both forearm orientation (supination vs. pronation) and loading conditions (passive, active, weighted) influence in vivo LHB tendon stiffness. However, the effect of forearm position appears to be most pronounced during active, low-load muscle contraction. Specifically, pronation showed a larger increase in stiffness from passive to active than supination, suggesting greater mechanical contribution of the LHB tendon during voluntary contraction in this position. In contrast, when an external load was applied, the influence of forearm position diminished, as both orientations showed comparable increases in stiffness under weighted conditions. The absence of positional differences under passive conditions likely reflects the minimal contribution of muscle/tendon tension when the muscle is relaxed. In contrast, under weighted conditions, the externally applied load appears to dominate tendon loading, reducing the relative influence of the forearm position. The transition from active to weighted conditions further supports this interpretation, as stiffness increased in both orientations but the magnitude of change was greater in supination, indicating that the relative contribution of external load may depend on baseline muscle involvement. Collectively, these findings suggest that forearm orientation primarily modulates muscle-driven tendon tension during low-load conditions, while external resistance becomes the dominant determinant of tendon loading during high mechanical demand. These directional sensitivities may have implications for rehabilitation and exercise strategies, as early-stage exercises performed under low-load conditions may be more sensitive to forearm position, while strengthening tasks involving external resistance may result in similar tendon loading regardless of forearm position. However, comparisons in which both forearm position and loading differed simultaneously should be interpreted as descriptive, as these comparisons do not isolate the independent effects of position or loading.

These outcomes provide practical guidance for rehabilitation strategies in the early stages of postoperative management following surgical interventions involving the LHB tendon [36,37,38]. The increase in tendon stiffness observed during active contraction in the pronated forearm position suggests that unrestricted active elbow flexion exercises performed in pronation may impose greater mechanical loading on the tendon compared with supinated positions. Accordingly, clinicians may consider limiting or avoiding early active exercises in pronation during the initial phases of rehabilitation when protection of the healing tendon is critical. On the other hand, exercises performed with the forearm in supination may represent a lower-loading alternative during early activation. Furthermore, the results from this study show that progressive external loading substantially increases tendon stiffness in both forearm positions, indicating that even modest resistance can significantly increase the mechanical demand on the tendon. Rehabilitation protocols may therefore benefit from a gradual progression of loading, beginning with low-demand movements and carefully introducing resistance as healing progresses. In addition to exercise-based rehabilitation, patients may also receive adjunct therapies aimed at controlling pain and supporting tissue healing [39,40]. Documenting these interventions is important to provide a comprehensive understanding of post-surgical care and to better interpret functional recovery and tendon loading patterns during rehabilitation. Importantly, SWE may provide a non-invasive method for monitoring tendon stiffness during recovery, potentially allowing clinicians to tailor rehabilitation programs according to patient-oriented loading responses.

Restoration of appropriate tendon tension during LHB tenodesis is critical for maintaining optimal shoulder biomechanics and minimizing postoperative complications. Alterations in tendon length, whether through shortening or lengthening during surgical reattachment, directly influence the muscle-tendon unit’s force-generating capacity by shifting its length–tension relationship. Prior studies have demonstrated that deviations from physiological tendon length can lead to sarcomere adaptation, either through the addition or subtraction of sarcomeres in series, in an attempt to re-establish equilibrium [23,25,27,28]. This adaptation may impair contractile efficiency by altering optimal actin–myosin overlap, ultimately reducing force production and joint stability [23,24,26]. These mechanical considerations are supported by clinical observations demonstrating the adverse effects of improper tensioning [15,41].

Biomechanically, increased LHB tendon stiffness under active conditions, as shown in this study, reflects elevated muscle-driven tendon tension and an amplified stabilizing role at the shoulder [15,25,41]. The increased stiffness observed in pronation during active contraction can suggest altered neuromuscular recruitment and increased mechanical demand in this orientation, which may influence movement strategies during early recovery. Pronation reorients the radial tuberosity, shifts the tendon’s line of action, and alters operating length and axial load transmission, collectively driving higher shear wave-measured stiffness [15,25,41]. While such adaptations may enhance stability, excessive or poorly timed tendon loading may compromise neuromuscular control and promote compensatory recruitment patterns [14,25], particularly in overhead athletes.

Several limitations should be considered when interpreting the findings of this study. First, the sample consisted of a relatively small and homogeneous group of 13 healthy young female participants, which substantially limits the generalizability of the results. The findings may not be directly applicable to males, older individuals, or patients with underlying shoulder pathology, particularly those undergoing treatment for Type II SLAP lesions. Sex- and age-related differences in muscle mass and neuromuscular activation patterns are known to affect musculotendinous mechanics and may influence LHB tendon stiffness. Consequently, the exclusive inclusion of female participants and the limited sample size warrant cautious interpretation of the results. The sample size was selected to provide an initial, exploratory characterization of in vivo LHB tendon stiffness using SWE; however, larger and more diverse cohorts are required to validate and extend these findings. Second, although SWE is a non-invasive and reproducible technique for assessing soft tissue stiffness, serving as an indirect surrogate for tendon tension, it is sensitive to operator technique and probe orientation. Measurements in this study were obtained following standardized protocols; nevertheless, appropriate training and strict adherence to acquisition methodology are critical for reproducibility when applying this technique more broadly. Third, measurements were obtained in a single shoulder position with changes in forearm orientation. Rehabilitation for type II SLAP lesions include flexion, external and internal rotation, and abduction strategies, among some. Finally, future studies should incorporate larger, sex-balanced cohorts across a wider age range and include individuals with confirmed SLAP pathology to improve clinical relevance and generalizability. Longitudinal SWE assessments before and after surgical intervention could further elucidate tendon and muscle adaptation during healing and rehabilitation. Such studies may help clarify how functional loading environments evolve over time and inform evidence-based rehabilitation progression. Ultimately, expanding normative datasets and validating SWE against clinical outcomes may support its future role as an adjunct tool for monitoring postoperative recovery.

## 5. Conclusions

This study characterized normative in vivo stiffness profiles of the long head of the biceps (LHB) tendon across various loading conditions and forearm orientations using ultrasound shear wave elastography. Tendon stiffness increased with muscle activation and external loading, with forearm position influencing stiffness primarily during active, low-load muscle contraction but not under passive or externally resisted conditions. These findings establish baseline in vivo stiffness patterns for the LHB tendon in asymptomatic individuals and provide a reference framework for future studies evaluating postoperative tendon mechanics following Type II SLAP lesion repair during early postoperative rehabilitation. Importantly, these data support the development of personalized rehabilitation strategies by enabling patient-specific assessments of tendon loading under functional conditions.

## Data Availability

The data presented in this study are available on request from the corresponding author.

## Abbreviations

The following abbreviations are used in this manuscript:

SWE	Shear Wave Elastography
LHB	Long Head of the Biceps
SLAP	Superior Labrum Anterior–Posterior
ROI	Regions of Interest
